# Contour Changes Following Immediate Placement of Ultra-Wide Implants in Molar Extraction Sockets without Bone Grafting

**DOI:** 10.3390/jcm9082504

**Published:** 2020-08-04

**Authors:** André Hattingh, Hugo De Bruyn, Manù Van Weehaeghe, Geert Hommez, Stefan Vandeweghe

**Affiliations:** 1 Private Practice for Periodontology and Oral Implantology, Sevenoaks, Kent TN15 8BQ, UK; achattingh@btinternet.com; 2 Oral Health Sciences, Faculty of Medicine and Health Sciences, Ghent University, 9000 Ghent, Belgium; Hugo.deBruyn@radboudumc.nl (H.D.B.); Manu.VanWeehaeghe@UGent.be (M.V.W.); Geert.hommez@ugent.be (G.H.); 3 Dental Faculty, Radboud University Medical Hospital, 6525EX Nijmegen, The Netherlands

**Keywords:** dental implant, immediate placement, ultra-wide diameter, extraction socket

## Abstract

The aim was to evaluate ridge reduction and mucosal recession following immediate placement of ultra-wide implants in molar sockets, without bone grafting. Impressions were taken prior to tooth extraction, 4 months and 1 year after implant placement. The casts were digitized and compared. Mucosal recessions and horizontal ridge reduction were measured. A total of 16 implants were in the maxilla and 11 in the mandible. At the buccal aspect, there was a mean reduction of 0.94 mm after 4 months and 0.89 mm after one year (*p* = 0.933). At the palatal/lingual aspect, this was 1.09 mm after 4 months and 0.69 mm after 1 year (*p* = 0.001). After 1 year, a recession of 0.59 mm was measured at the zenith, 1.04 mm at the mesial and 0.98 mm at the distal papilla. The mean midfacial horizontal ridge reduction was 1.23 mm after 4 months and 1.45 mm after 1 year. At the midpalatal/midlingual aspect, the mean horizontal reduction was 1.43 mm after 4 months and 1.16 mm after 1 year. Immediate implant placement without bone grafting in the posterior jaw yields a significant horizontal ridge reduction and minor mucosal recession. Clinicians should anticipate the amount of ridge reduction and consider augmentation at the time of implant placement.

## 1. Introduction

Alveolar bone develops in relation to tooth eruption. It is well recognized that as a result of tooth removal, alveolar bone undergoes atrophic changes that result in horizontal and vertical reduction of crestal dimensions due to loss of the periodontal ligament and resorption of bundle bone in the tooth socket [1,2,3]. These changes lead to reduced bone volume for the potential placement of dental implants and might create both functional and aesthetic challenges during the restorative treatment [4]. It is essential to have sufficient bone volume and a favorable anatomy within the alveolar ridge to allow for the desired aesthetic and functional reconstruction, following implant placement [1].

Most of the inevitable alveolar socket remodeling [1,3,5] following tooth removal, occurs within the first 6 months [1], but further resorption of bone continues progressively albeit at a slower rate throughout life, leading to further changes in the anatomy of both jaws [6,7,8]. This process reduces the horizontal and vertical dimensions of the edentulous ridge, but the buccal aspect is affected to a greater degree [3,9,10]. Consequently, a shorter and narrower ridge follows [11,12], and this effect contributes to a more palatal/lingual movement of the ridge [13]. Implant placement and survival may become less ideal because of these alterations [5]. However, there are other factors that could also play a role in the residual amount of bone volume, after tooth extraction. These could involve trauma to the surrounding hard and soft tissues during tooth removal [14] or a reduction in alveolar bone because of periodontal or peri-apical pathology. Soft tissue elevation could also have a negative impact on bone preservation, and it is suggested that a flapless surgical approach could reduce the amount of bone loss [15,16,17,18]. It is assumed that alveolar bone remodeling is thus affected by various simultaneous factors, and its magnitude is patient, site and time dependent [19].

To compensate for these inevitable dimensional changes, implant placement can be considered at the time of tooth removal, before ridge reduction has commenced [20,21]. This would result in less bucco-lingual bone reduction and a wider crest compared to delayed implant placement [22].

Although immediate implant placement does not guarantee a reduced amount of bone remodeling post-extraction [19,23], it has the advantage of a single surgical procedure, reduced cost to the patient, ideal implant positioning [24,25], preservation of bone in the extraction site [26,27], and a shortened treatment time [28].

Another solution would be to delay implant placement until healing of the extraction socket has occurred [28,29]. In this case, socket preservation would be recommended in order to maintain as much of the bony dimensions as possible [30,31].

Although many studies have reported on the dimensional changes following tooth removal and implant placement in the aesthetic zone [32,33,34], to our knowledge, none are available solely on molar sites. Immediate implant placement in molar sites is predictable in terms of bone loss and implant survival [35,36,37], but little is known about the aesthetics and soft tissue alterations. This is an important issue since numerous studies report compromised results in the aesthetic region when immediate implant placement protocols are used [38,39]. Most of these aesthetic compromises relate to visible volume changes that can be seen post treatment.

The aim of this prospective study was to evaluate the bucco-lingual/palatal dimensional changes around ultra-wide implants immediately placed in molar sockets.

## 2. Experimental Section

### 2.1. Clinical Procedure

All patients were treated by one periodontist (AH) and were part of a prospective clinical study. When acute peri-apical or periodontal infections were absent, and no obvious bony defects were detected, patients were considered eligible for immediate implant placement and asked to sign an informed consent in accordance with guidelines approved by the Ethical Committee of the Health Research Authority, England. The study was approved by London—Camden and Kings Cross Research Ethics Committee, REC reference number 15/LO/1099. Patients were provided with a written treatment plan, and upon receipt of their consent to treat, the associated molar was scheduled for removal and implant placement.

In total 51 patients were treated, but only the last 34 included patients were part of the volumetric analyses due to delays in the study setup.

From these 34 patients, an alginate impression was taken prior to tooth removal and poured in white orthodontic gypsum (Crystacal R Plaster, Saint-Gobain GmbH, Aachen, Germany) within 1 h to obtain a study cast (T0). This served as the baseline reference.

A detailed description of the surgical protocol was published earlier as a case report [40]. In brief, the molar was sectioned before removal, thereby taking care that no bone was ever removed in the process, and no soft tissue flaps were raised. Osteotomy preparation was completed, followed by implant insertion (MAX^®^, Southern Implants, Irene, South Africa). A healing abutment was connected to the implant, and soft tissue adaptation as well as void closure were obtained with suturing and hemostatic collagen sponge. The last mentioned was only placed at the level of the healing abutment. None of the cases received a bone graft, regardless of the gap distance between the implant and the inner wall of the socket.

Patients were asked not to rinse for 1 week after surgery and were given a prophylactic antibiotic as well as analgesics.

After 4 months, reverse torque testing (30Ncm) was performed to confirm implant integration and a second alginate impression (T1) was taken. Patients returned to their referring practitioners for completion of the restorative phase of treatment and recalled for assessment on a yearly basis.

At the 1-year follow-up, a clinical and radiographic examination was performed, and a third alginate impression was taken (T2).

**Figure 1:**
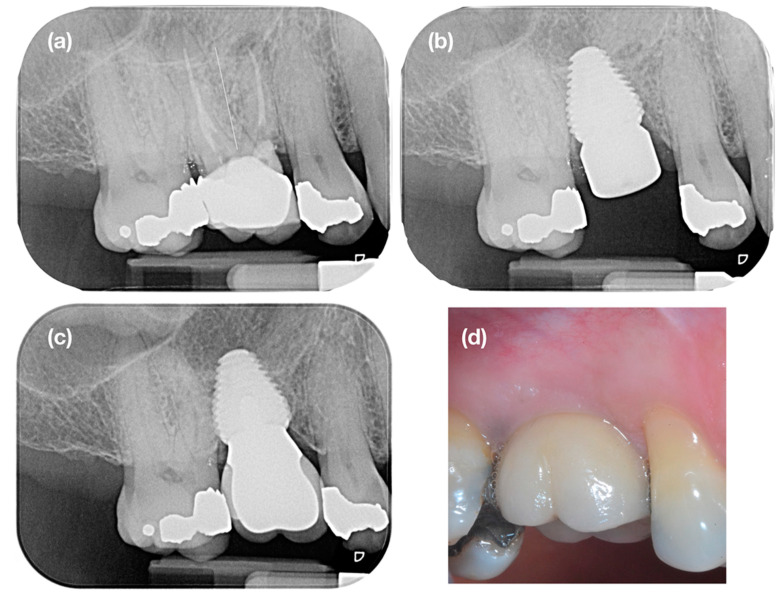
Case example: (**a**) tooth 16 was extracted because of recurring peri-apical pathology and (**b**) immediately replaced by an ultra-wide diameter implant (8 × 11 mm), which was positioned 2 mm subcrestal to compensate for post-extraction bone resorption. One year later (**c**), complete bone fill can be observed, and the bone level has stabilized at the implant neck. (**d**) A small mucosal recession and a slight concave profile of the buccal mucosa is noticeable.

The clinical outcome has been reported in a separate publication [37]. A bone increase of 0.15 mm was reported after a follow-up of 23 months (Figure 1). There were no significant differences in terms of probing depth and bleeding on probing compared to the contra-lateral tooth. Overall, patient satisfaction was high, which demonstrates that this treatment modality is well accepted.

### 2.2. Data Analyses

All casts were digitized using an intra-oral scanner (Trios2, 3Shape, Copenhagen, Denmark) and saved as an open format STL file.

**Figure 2:**
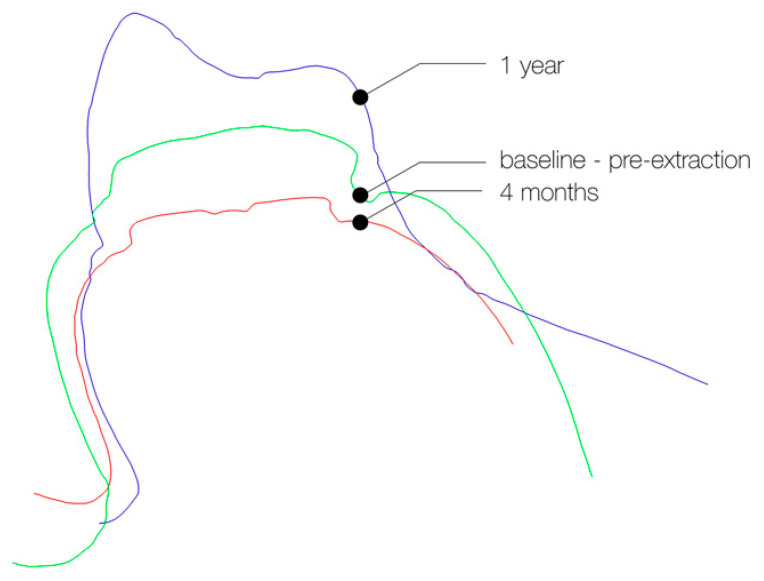
Cross-section of the superimposed casts, taken at 3 different time-points. The green line (=baseline) represents the contour of the hopeless tooth before extraction. The red line depicts the contour of the healing abutment, 4 months after implant placement. The purple line represents the contour of the implant supported restoration at 1 year.

**Figure 3:**
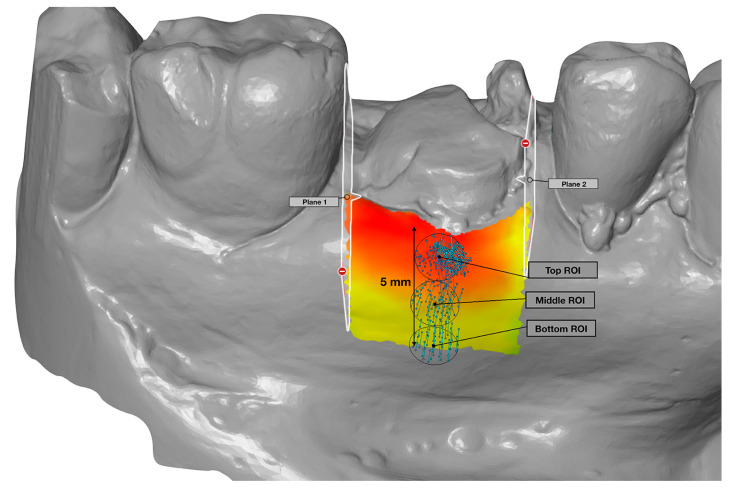
In the buccal and palatal/lingual mucosa, an area was selected defined by 2 planes (mesial and distal), which are elongations of the contact surface of the teeth and at the apical side by a 5 mm distance from the mucosal margin. This area served as the reference to determine the horizontal reduction. In addition, 3 circular regions of interest (Top, Middle and Bottom ROI), with a diameter of 2 mm, were created on the midfacial axis, to measure the midfacial dimensional changes.

Using metrology software (Geomagic Qualify, 3D Systems, Rock Hill, SC, USA), the virtual casts from baseline, 4 months and 1 year were superimposed using a best-fit algorithm (Figure 2). At the mid-facial and mid-palatal/lingual aspect, 3 reference points (1 mm, 3 mm and 5 mm apically of the mucosal margin) were marked as center points of 2 mm-diameter circles [34]. These 3 circles on the buccal and palatal/lingual mucosa were defined as the top region of interest (ROI), middle ROI and bottom ROI (Figure 3).

The aligned casts were now loaded in another metrology software package (GOM Inspect 2019, GOM GmbH, Braunschweig, Germany) for additional measurements. Reference points were placed at the zenith and top of the papillae on the casts from baseline and 1 year follow up. These distances were recorded to determine recession of the zenith or papillae.

On the buccal and palatal/lingual mucosa of the baseline cast, an area was selected, defined at the coronal side by the mucosal margin, at the mesial and distal side by 2 planes that are elongations of the contact surface of the teeth and at the apical side by a 5 mm distance from the mucosal margin (Figure 3). Within this area, the shortest distance to the 4 months and 1-year cast were measured to determine the reduction in thickness between the given time points.

Statistical analyses were done using SPSS v22 (IBM Corporation, Armonk, NY, USA). Mann–Whitney U-test was used for comparison between upper and lower jaw. Wilcoxon signed rank test was used for the comparisons between time points. The level of significance was set at *p* < 0.05.

A high degree of intra-examiner reliability was found between 30 measurements. The average measure ICC was 0.929 with a 95% confidence interval from 0.845 to 0.968 (F (26,26) = 13.824, *p* < 0.001). A high degree of inter-examiner reliability was found between 30 measurements. The average measure ICC was 0.912 with a 95% confidence interval from 0.807 to 0.960 (F (26,26) = 11.177, *p* < 0.001).

## 3. Results

**Figure 4:**
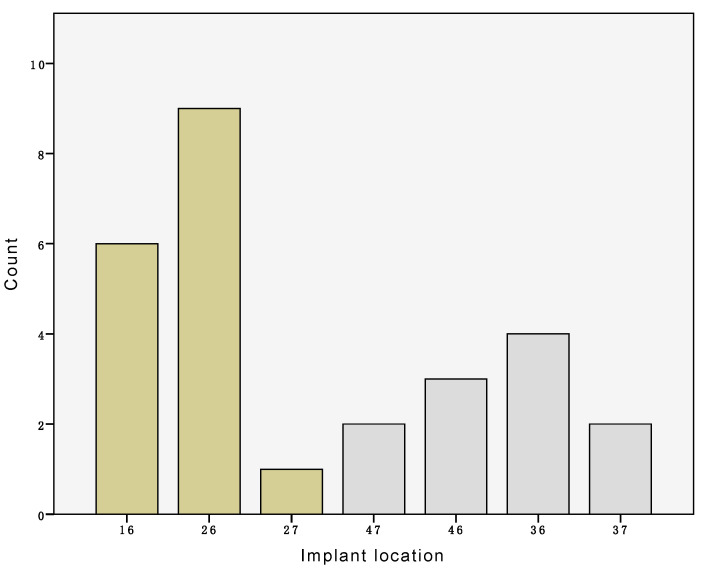
Overview of the number of implants per location.

Seven cases were excluded because of irregularities in one of their casts. In four casts, voids in the impression lead to deformation of the cast at the gingival margin. In three other cases, the borders of the impressions were short and had not reached the vestibulum, resulting in a smaller buccal region than that defined adequate to perform the measurements. Finally, 27 sets of three models derived from 20 male and 7 female patients with a mean age 61 years (SD 9, range 37–80) remained. A total of 16 implants were located in the maxilla and 11 in the mandible. An overview of their exact location is given in Figure 4.

At the buccal aspect, there was a mean reduction of 0.94 mm (SD 0.67, range −0.39–2.16) between baseline and 4 months and 0.89 mm (SD 0.78, range −1.54–2.11) between baseline and one year. Dimensional alterations between 4 months and one year were not statistically significant (*p* = 0.933).

**Figure 5:**
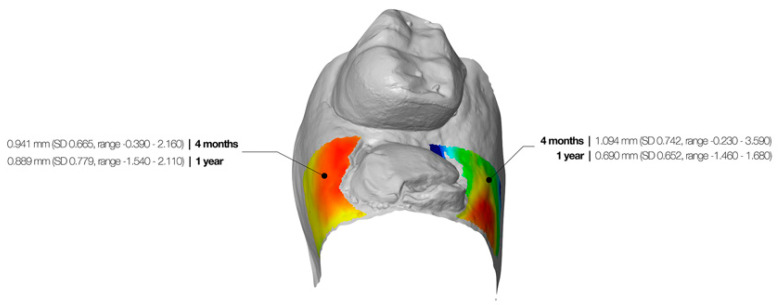
Graphic representation of the colormap, which represents the horizontal changes. The dimensional alterations after 4 months and 1 year are depicted in the figure.

At the palatal/lingual aspect, this was 1.09 mm (SD 0.74, range −0.23–3.59) between baseline and 4 months and 0.69 mm (SD 0.65, range −1.46–1.68) between baseline and 1 year (Figure 5). Dimensional alterations between 4 months and one year were statistically significant (*p* = 0.001). However, no significant changes were found between the maxilla and mandible (*p* > 0.050).

The zenith point receded with a mean value of 0.59 mm (SD1.37, range −2.71–3.33) between baseline and 1 year. Advanced recession (>1 mm) was observed at 44.4% of the restorations. The mesial papilla receded 1.04 mm (SD 0.52, range −0.64–1.91) and the distal 0.98 mm (SD 0.68, range −1.20–1.99) during the same period.

The mean midfacial ridge reduction was 1.23 mm (SD 0.71, range −0.09–3.14) after 4 months and 1.45 mm (SD 0.70, range 0.18–3.09) after 1 year. At the midpalatal/midlingual aspect, the mean reduction was 1.43 mm (SD 0.74, range 0.34–3.75) after 4 months and 1.16 mm (SD 0.67, range 0.23–3.13) after 1 year.

An overview of the dimensional alterations per region at the midfacial and midpalatal/lingual aspect is depicted in Table 1.

## 4. Discussion

Following tooth extraction, bone remodeling occurs as part of the healing process and the loss of bundle bone [3,13]. This may hamper implant placement especially in the posterior maxilla where a significant reduction in vertical ridge dimensions occurs, in combination with pneumatization of the sinus [41,42]. Farina et al. [5] reported a more apical position of the ridge, a lower bone width and a more coronal position of the sinus floor compared to dentate sites in the maxilla. In the mandible, a reduced bone height and width was observed compared to the dentate areas [43]. In some cases, the residual ridge volume after healing is insufficient to place dental implants without bone augmentation procedures. Therefore, it might seem wise to anticipate collapse of the bone ridge and to consider placing the implant immediately after tooth removal.

In molar extraction sockets, immediate implant placement is very much determined by the anatomy of the socket. Conventional diameter implants often rely on the interradicular bony septum to achieve primary stability. However, in cases where the septal bone is thin or absent, a wide diameter implant which engages some of the inner aspects of the socket walls would be more suitable to provide primary stability [44]. This could explain why ultra-wide diameter implants demonstrate survival rates of over 95% when used in this situation [35,36,37]. However, it will not prevent the initial bone remodeling, and the question remains as to how it will affect the outcome of the implant in the long term.

Even though immediate implants demonstrate good clinical survival, the aesthetic outcome is often inferior compared to a delayed approach, including socket preservation [45]. In a systematic review, Cosyn et al. [46] reported that most of their immediate cases demonstrated mid-facial recession. In the approximal area, however, there was a low risk for recession, with 70% of the cases demonstrating complete papilla fill [47,48]. This is probably because no flaps were raised, which prevents disruption of the papillae. It also contributes to the maintenance of blood supply, which is crucial to limit bone resorption [49].

Case selection is crucial to assure a predictable result. A thin gingival biotype resulted in a mid-facial recession for 85% of the patients, with a mean tissue thickness of 1.5 mm. In comparison, a thick biotype resulted in a mid-facial recession in 38% of the patients with an overall mean thickness of 0.56 mm [50,51]. On the other hand, Chappuis et al. [52] evaluated the dimensional alterations 2 months after extraction and found a significant increase in mucosal thickness within thin biotypes. However, the overall dimensions decreased due to bone resorption. Another study reported a vertical mid-facial bone loss of 62.3% or 7.5 mm, which resulted in a 1.6 mm mid-facial recession [53]. In our study we found a mid-facial recession of 0.59 mm, which is lower than the afore mentioned studies. This positive outcome could be related to the fact that extraction and implant placement were done with a flapless approach. Hence, the mucoperiosteum was not disrupted. Not raising flaps and not disrupting the blood flow in the bundle bone led to minimal trauma and may account for the outcome. We must point out that at some teeth, minor soft-tissue defects were present before extraction due to defects associated with the inoperative tooth. In those cases (22.2%), an improvement in the mucosal level was noticed, which may have a positive effect on the minimal recession found after 4 and 12 months.

When it comes to the dimensional changes, it has been shown that most of the remodeling takes place in the mid-facial region [53]. In a one-year follow-up study of immediate implants in the aesthetic region, Cardaropoli et al. [54] reported 0.69 mm horizontal bucco-palatal ridge reduction in a grafted group and 1.92 mm in a non-grafted group. In our study, the horizontal reduction was 2.61 mm, which is significantly higher and can be explained by the fact that no bone graft was used, and only molar sites were included. The latter have larger sockets, extraction may be more difficult to perform, and hence, more bone resorption can be expected.

When it comes to time of implant placement, Arora and Ivanovski found a similar horizontal buccal ridge resorption for immediate (0.61 mm) and delayed (0.72 mm) implant placement [34]. Tian et al. [55] reported a mean buccal horizontal reduction of 0.62 mm, 1 year after immediate implant placement and grafting of the residual jump gap combined with immediate provisionalisation. These figures are lower compared to the results of our study (0.889 mm) but can be explained by the absence of bone grafting and the difference in socket anatomy and size (molar vs anterior). Most of the afore mentioned literature relates to the anterior maxilla, where the labial bone thickness is thin. However, Theye et al. [56] measured the bony dimensions at first molar sites and found that 20.8% of mandibular and 62.18% of maxillary first molars have a buccal cortical wall of less than 1 mm thick.

The occurrence of midfacial mucosal recession has been reported at immediate as well as delayed implants [57,58,59]. Although this recession is commonly earlier observed at immediate implants, mucosal recession also occurs in the long run, resulting in little difference between the two treatment approaches [60]. In the anterior maxilla, Arora and Ivanovski [34] reported 0.26 mm recession at immediate implants, of which 27% demonstrated advanced recession (>1 mm). This is lower in comparison to our findings, where 0.59 mm of recession and 44.4% of advanced recessions were observed. However, in the study by Arora and Ivanovski [34], the jump distance was filled with a bone graft, which may have attributed to better preservation of the hard and soft tissues. In the posterior jaw, Checchi et al. [61] compared immediate ultra-wide implants with conventional diameter implants in grafted molar sockets. After 1 year, the aesthetic outcome was significantly better for the delayed approach. Despite small but significant recessions around immediate implants, there are obvious advantages related to this treatment protocol, such as shortened treatment, a reduced number of interventions and a high patient satisfaction [62,63].

Tian et al. [55] also reported a minor increase in contour from 6 to 12 months and contributed this recovery to the delivery of the definitive restoration and the increased volume in the papilla regions. Moreover, in our study, a small increase in ridge dimensions was found between 4 months and one year. Although bone resorption and a decrease in hard-tissue volume is inevitable, some authors have reported an increase in soft-tissue thickness after tooth extraction, especially in thin biotypes [64,65]. The authors attribute this to the healing process following tooth removal.

Although our measurements were done digitally, the information was based on gypsum casts, produced from alginate impressions. A better approach would have been to make digital impressions, but an intra-oral scanner wasn’t available in the practice at the time of the study. The direct digitization of the dentition by means of an intra-oral scanner is at least as accurate as the indirect digitization of gypsum study casts using a lab scanner [66,67]. The use of an intra-oral scanner could have improved the precision and would have simplified the protocol [68,69]. Moreover, it has been reported that the use of digital measurements, by comparing superimposed virtual casts, is more reliable and consistent compared to conventional measurements [70,71,72,73].

In this study, ridge alterations were measured by comparing the mucosal outline at different time points. But, this approach does not provide precise data about the crestal bone dimensions. One can only assume that changes in the mucosal outline will be a result of dimensional alterations of the soft-and hard tissues. Ideally, changes in crestal bone volume could be determined by comparing cone-beam CT scans and adding these images as an additional layer to determine the soft-tissue thickness. This would, however, expose the patient to additional radiation and was therefore not allowed by the ethical committee.

The findings from this study demonstrate that immediate implant placement, using ultra-wide diameter implants and a flapless approach, is a successful treatment method. However, clinicians should also be aware that bone remodeling and minor mucosal recessions will occur and, therefore, should consider placing the implant subcrestally and using a bone graft to limit resorption and avoid aesthetic problems [74].

## 5. Conclusions

This clinical study clearly showed that immediate placement of an ultra-wide diameter implant in molar extraction sockets, using an atraumatic extraction technique without raising flaps, leads to an acceptable aesthetic outcome in terms of minimal contour changes as well as stability over time. Significant ridge reduction occurred during the first four months after extraction and clinicians should therefore consider bone augmentation at time of implant placement to reduce bone resorption.

## Figures and Tables

**Table 1:** Overview of the ridge reduction at the buccal and palatal/lingual aspect of the implant site. Numbers represent the mean value, standard deviation and range.

	4 Months	12 Months	
**Buccal horizontal reduction**			
	0.94 mm (SD 0.67, range −0.39–2.16)	0.89 mm (SD 0.78, range −1.54–2.11)	*p* = 0.933
**Buccal midfacial reduction**			
Top 2 mm ROI	1.53 mm (SD 0.96, range −0.42–3.85)	1.43 mm (SD 0.74, range 0.05–3.23)	*p* = 0.195
Middle 2 mm ROI	1.31 mm (SD 0.73, range −0.09–2.79)	1.59 mm (SD 0.81, range 0.09–3.15)	*p* = 0.010
Bottom 2 mm ROI	0.86 mm (SD 0.62, range 0.10–2.78)	1.33 mm (SD 0.76, range −0.23–2.88)	*p* = 0.001
Overall	1.23 mm (SD 0.71, range −0.09–3.14)	1.45 mm (SD 0.70, range 0.18–3.09)	*p* = 0.013
**Palatal/Lingual horizontal reduction**			
	1.09 mm (SD 0.74, range −0.23–3.59)	0.69 mm (SD 0.65, range −1.46–1.68)	*p* = 0.001
**Palatal/Lingual midfacial reduction**			
Top 2 mm ROI	1.68 mm (SD 0.81, range 0.48–3.62)	1.18 mm (SD 0.81, range −0.32–2.97)	*p* = 0.010
Middle 2 mm ROI	1.51 mm (SD 0.73, range 0.22–3.62)	1.35 mm (SD 0.72, range 0.30–3.32)	*p* = 0.186
Bottom 2 mm ROI	1.11 mm (SD 0.92, range 0.20–4.02)	0.97 mm (SD 0.68, range 0.09–3.10)	*p* = 0.302
Overall	1.43 mm (SD 0.74, range 0.34–3.75)	1.16 mm (SD 0.67, range 0.23–3.13)	*p* = 0.044
